# Uniform FDG-PET guided GRAdient Dose prEscription to reduce late Radiation Toxicity (UPGRADE-RT): study protocol for a randomized clinical trial with dose reduction to the elective neck in head and neck squamous cell carcinoma

**DOI:** 10.1186/s12885-017-3195-7

**Published:** 2017-03-21

**Authors:** Sven van den Bosch, Tim Dijkema, Martina C. Kunze-Busch, Chris H. J. Terhaard, Cornelis P. J. Raaijmakers, Patricia A. H. Doornaert, Frank J. P. Hoebers, Marije R. Vergeer, Bas Kreike, Oda B. Wijers, Wim J. G. Oyen, Johannes H. A. M. Kaanders

**Affiliations:** 1 Department of radiation oncology, Radboud University Medical Center, huispost 874, P.O. Box 9101, Nijmegen, 6500 HB The Netherlands; 2 Department of radiation oncology, University Medical Center Utrecht, Utrecht, The Netherlands; 3 Department of radiation oncology (MAASTRO), Research Institute GROW, Maastricht University, Maastricht, The Netherlands; 4 Department of radiation oncology, VU University Medical Center, Amsterdam, The Netherlands; 5 Department of radiation oncology, Radiotherapiegroep, Arnhem, The Netherlands; 6 Department of radiation oncology, Radiotherapeutisch Instituut Friesland, Leeuwarden, The Netherlands; 7 Department of nuclear medicine, Radboud University Medical Center, Nijmegen, The Netherlands; 8 The Institute of Cancer Research and The Royal Marsden NHS Foundation Trust, London, UK

**Keywords:** Head and neck cancer, Squamous cell carcinoma, Accelerated radiation therapy, Dose reduction, Dose de-escalation, Elective nodes, FDG-PET, Euality of life

## Abstract

**Background:**

In definitive radiation therapy for head and neck cancer, clinically uninvolved cervical lymph nodes are irradiated with a so-called ‘elective dose’ in order to achieve control of clinically occult metastases. As a consequence of high-resolution diagnostic imaging, occult tumor volume has significantly decreased in the last decades. Since the elective dose is dependent on occult tumor volume, the currently used elective dose may be higher than necessary.

Because bilateral irradiation of the neck contributes to dysphagia, xerostomia and hypothyroidism in a dose dependent way, dose de-escalation to these regions can open a window of opportunity to reduce toxicity and improve quality of life after treatment.

**Methods:**

UPGRADE-RT is a multicenter, phase III, single-blinded, randomized controlled trial.

Patients to be treated with definitive radiation therapy for a newly diagnosed stage T_2-4_ N_0-2_ M_0_ squamous cell carcinoma of the oropharynx, hypopharynx or larynx are eligible. Exclusion criteria are recurrent disease, oncologic surgery to the head and neck area, concomitant chemotherapy or epidermal growth factor receptor inhibitors.

In total, 300 patients will be randomized in a 2:1 ratio to a treatment arm with or without de-escalation of the elective radiation dose and introduction of an intermediate dose-level for selected lymph nodes. Radiation therapy planning FDG-PET/CT-scans will be acquired to guide risk assessment of borderline-sized cervical nodes that can be treated with the intermediate dose level.

Treatment will be given with intensity-modulated radiation therapy or volumetric arc therapy with simultaneous-integrated boost using an accelerated fractionation schedule, 33 fractions in 5 weeks. The primary endpoint is ‘normalcy of diet’ at 1 year after treatment (toxicity). The secondary endpoint is the actuarial rate of recurrence in electively irradiated lymph nodes at 2 years after treatment (safety).

**Discussion:**

The objective of the UPGRADE-RT trial is to investigate whether de-escalation of elective radiation dose and the introduction of an intermediate dose-level for borderline sized lymph nodes in the treatment of head and neck cancer will result in less radiation sequelae and improved quality of life after treatment without compromising the recurrence rate in the electively treated neck.

**Trial registration:**

ClinicalTrials.gov Identifier: NCT02442375.

## Background

In definitive radiation therapy for head and neck cancer, generally two dose-levels are delivered.

A high dose, the so-called “boost dose” to eradicate macroscopic tumor and a lower dose, the so-called “elective dose” to achieve control of clinically occult metastases in cervical lymph nodes. The current equivalent dose in 2 Gy fractions (EQD2) prescribed to the elective volume is 45–50 Gy and is based on literature from the nineteen-fifties [1]. At that time, assessment of the neck only consisted of physical examination due to the lack of sufficiently sensitive diagnostic imaging of lymph nodes.

Today, ultra-sound (US) with fine needle aspirated cytology (FNAC), computed tomography (CT) and magnetic resonance imaging (MRI) provide high resolution anatomical detail and have a high sensitivity and specificity in the detection of cervical lymph node metastases in head and neck cancer [2], even in a neck without palpable lymph nodes [3].

As a consequence of the implementation of high-resolution diagnostic imaging techniques, tumor deposits measuring only a few millimeters are now detected and added to the boost volume. It is therefore plausible that nowadays occult tumor volume in radiologically uninvolved lymph nodes is much smaller than in the era before the implementation of these imaging techniques. However, the radiation therapy dose prescription practice for elective nodal regions has not changed over the years.

Since the dose required to control subclinical disease is dependent on occult tumor volume [4, 5], it would make sense to refine the traditional binary dose prescription to a more gradual one that is proportional to tumor volume. For this purpose, molecular imaging using fluorodeoxyglucose positron emission tomography (FDG-PET) can improve the accuracy of current diagnostic imaging assuming that FDG-uptake represents tumor cell density, or at least is a good surrogate for this [6].

The potential value of FDG-PET imaging in the management of cervical lymph nodes lies in the decision-making process whether borderline-sized nodes should be treated with a boost or elective dose [7]. It is even conceivable that borderline-sized nodes having mild FDG-uptake may not require the maximum dose since they are likely to contain no or only a small tumor volume. An intermediate dose level may be sufficient for such nodes.

Because bilateral irradiation of cervical lymph node regions contributes to dysphagia, xerostomia and thyroid dysfunction in a dose dependent way [8, 9], dose reduction to these regions can open a window of opportunity to reduce toxicity and improve quality of life after treatment [10].

A treatment planning study performed at the Radboudumc showed that de-escalation of the elective dose as proposed in this study protocol, can reduce the dose to normal tissues such as the swallowing musculature, thyroid- and salivary glands (unpublished data). Normal tissue complication probability models from the available literature [11–15] showed that a relevant decrease in toxicity can be expected for xerostomia (absolute up to 14%, relative risk up to 39%), dysphagia (absolute up to 12%, relative risk up to 67%) and hypothyroidism (absolute up to 20%, relative risk up to 50%).

Given these considerations we believe that the traditional binary dose prescription in head and neck cancer is outdated. In this trial, a more gradual dose prescription will be used with de-escalation of the elective radiation dose and the introduction of an intermediate dose-level in the treatment of head and neck cancer. The aim is to investigate whether such a treatment will result in less radiation sequelae and improved quality of life after treatment (expressed as a normalcy of diet) without compromising the recurrence rate in electively irradiated lymph nodes.

## Methods/Design

### Objectives

To determine whether a more gradual dose prescription with de-escalation of the elective radiation dose and the introduction of an intermediate dose-level in the treatment of head and neck cancer will result in less radiation sequelae and improved quality of life after treatment (toxicity) without compromising the recurrence rate in electively irradiated lymph nodes (safety).

### Study design

This is a multicenter, phase III, single-blinded, randomized controlled trial.

Treatment allocation at randomization will be at a ratio of 2:1 in favor of the intervention arm. Randomization will be balanced for institution, tumor-site, T- and N-stage, and human papillomavirus status using minimization with a random element. Randomization and clinical trial data management is provided by the IKNL clinical research department.

**Fig. 1:**
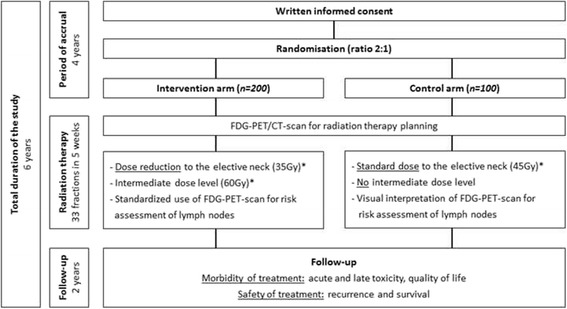
Flow chart giving an overview of the study design. *the reported dose is the equivalent dose in 2 Gy fractions (EQD2)

A flow chart giving an overview of the study design is shown in Fig. 1.

### In- and exclusion criteria

Adult patients having a new, pathologically proven squamous cell carcinoma located in the larynx, oropharynx or hypopharynx with stage T_2-4_ N_0-2_ M_0_ are eligible for inclusion after written informed consent. Patients must be able to undergo accelerated radiation therapy and have a World Health Organization performance status of 0–2.

Main exclusion criteria are concomitant chemotherapy or epidermal growth factor receptor inhibitors for this tumor, prior anticancer treatment to the head and neck area (surgery, chemotherapy or radiation therapy), previous malignancies or uncontrolled diabetes mellitus.

### Endpoints

Primary endpoint (toxicity): ‘normalcy of diet’ at 1 year after treatment, measured using the performance status scale for patients with head and neck cancer (PSS-HN) [16].

Secondary endpoint (safety): actuarial rate of recurrence in electively irradiated lymph nodes at 2 years after treatment.

Other endpoints:

Acute toxicity (mucositis, dysphagia and skin reaction).

Late toxicity (with focus on xerostomia, dysphagia and hypothyroidism).

Quality of life (general-, xerostomia- and dysphagia related quality of life).

Recurrence (local, regional, loco-regional and distant).

Survival (overall, disease specific and disease free).

### Sample size calculation

This study was designed to detect a 10-point difference on the PSS-HN ‘normalcy of diet’ score at 12 months after radiation therapy with a power of 90% at a two-sided significance level of 0.05. An average ‘normalcy of diet’ score of 70 is expected after standard treatment. To achieve this significance level with an unequal randomization ratio (2:1), a total of 300 patients needs to be included.

The current rate of recurrence in electively irradiated lymph nodes was estimated to be 5% at 2 years after treatment [17]. An equal rate of recurrence is expected in the intervention arm, despite elective dose de-escalation. A recurrence rate of ≥10% will be considered clinically relevant and unacceptable. This difference can be detected with the number of patients planned for the primary outcome of the study and a one-sided α = 0.10.

The total duration of this trial is estimated to be 6 years (4 years accrual, 2 years follow-up).

### Pre-treatment evaluation

Pre-treatment evaluation will include physical examination and flexible endoscopy of the upper aerodigestive tract, biopsy of the tumor, MRI and/or CT-scan of the head and neck area, and US of the neck including FNAC of cervical lymph nodes. All patients are evaluated by a multidisciplinary head-and-neck oncology team.

### Radiation therapy planning FDG-PET/CT-scan

In order to ensure that in the multicenter setting of this trial, the acquired quantitative data and standardized uptake value (SUV) recoveries are interchangeable between study sites, all FDG-PET/CT-scans will be acquired on EARL accredited scanners (http://earl.eanm.org) following the European Association of Nuclear Medicine (EANM) procedure guidelines for tumor PET imaging v2.0 [18].

FDG-PET/CT-scans of the head and neck area will be acquired in radiation therapy treatment position using a custom-made thermoplastic head, neck and shoulders mask to immobilize the patient during radiation therapy and the scanning procedures. A diagnostic CT-scan using an intravenous contrast agent will be acquired in one session on the PET/CT-scanner for treatment planning.

### Standardized uptake ratio (SUR)

In this trial, the SUV will be normalized by an internal image-derived standard, in order to minimize inter-study variability of FDG-uptake [18]. The cervical spinal cord will be used as the internal image-derived standard [19].

The SUR will be calculated using the following formula:

SUR_max_ = (SUV_max_in region of interest/SUV_mean_of internal standard ).

### Risk assessment of lymph nodes

#### Intervention arm

In the intervention arm, lymph nodes will be classified into three risk-levels and will be treated with a corresponding radiation dose. The FDG-PET-scan will guide risk assessment using standardized methods in order to minimize inter-institutional and inter-operator variations.

- *High-risk (macroscopic tumor)*: comprises metastatic nodes that will be identified by (1) positive cytology or (2) necrosis on imaging or (3) SUR_max_ ≥ 2.0.

- *Intermediate-risk*: comprises lymph nodes of borderline size having intermediate FDG-uptake and will be identified by (1) summed long- and short-axis diameter ≥ 17 mm and (2) SUR_max_ ≥ 1.5 and <2.0 and (3) cannot be high-risk (e.g. positive cytology or necrosis on imaging). These criteria are based on an in-depth risk assessment on recurrence in 1166 electively irradiated nodes in 264 patients. Not overtly pathologic lymph nodes with a summed diameter ≥ 17 mm had an increased risk to recur after elective treatment (Hazard Ratio: 17.8, 95%CI: 5.7–55, *p* < 0.001) [17].

- *Low-risk (microscopic tumor)*: comprises elective lymph node regions defined in the protocol based on tumor site and stage and will be delineated according to published guidelines [20].

Retropharyngeal lymph nodes will be evaluated by traditional means as standardized FDG-PET-guided risk-assessment is not applicable to these nodes.

#### Control arm

In the control arm, no areas of intermediate risk will be identified. Lymph nodes are either assigned to the high-risk (macroscopic) volume or low-risk (elective) volume by traditional means (positive cytology or necrosis on imaging or short-axis diameter ≥ 10 mm, ≥11 mm for subdigastric nodes).

The FDG-PET-scan may be used in the decision making process by means of visual interpretation.

### Delineation and margins

For the primary tumor, the gross target volume (GTV_p_) will be delineated by traditional means using information from clinical examination and diagnostic imaging (CT and/or MRI) and will encompass all overtly macroscopic disease. In both treatment arms, a biological target volume (BTV_p_) of the primary tumor is created by means of adaptive threshold iso-contouring based on the FDG-SUR maps [19]. Gross / high-risk lymph node metastases will be delineated separately (GTV_n_).

A clinical target volume (CTV) is created to cover all routes of potential subclinical disease spread.

The GTV_p_ will be expanded by a 3D margin of 10 mm and the GTV_n_ will be expanded by a 3D margin of 5 mm (10 mm in case of extra nodal disease on imaging) in order to create the CTV_high-risk_. The CTV_high-risk_ will be adjusted for anatomical borders in which microscopic disease is unlikely to extend. No CTV expansion will be used for intermediate-risk lymph nodes as extra nodal disease is unlikely in these nodes.

**Fig. 2:**
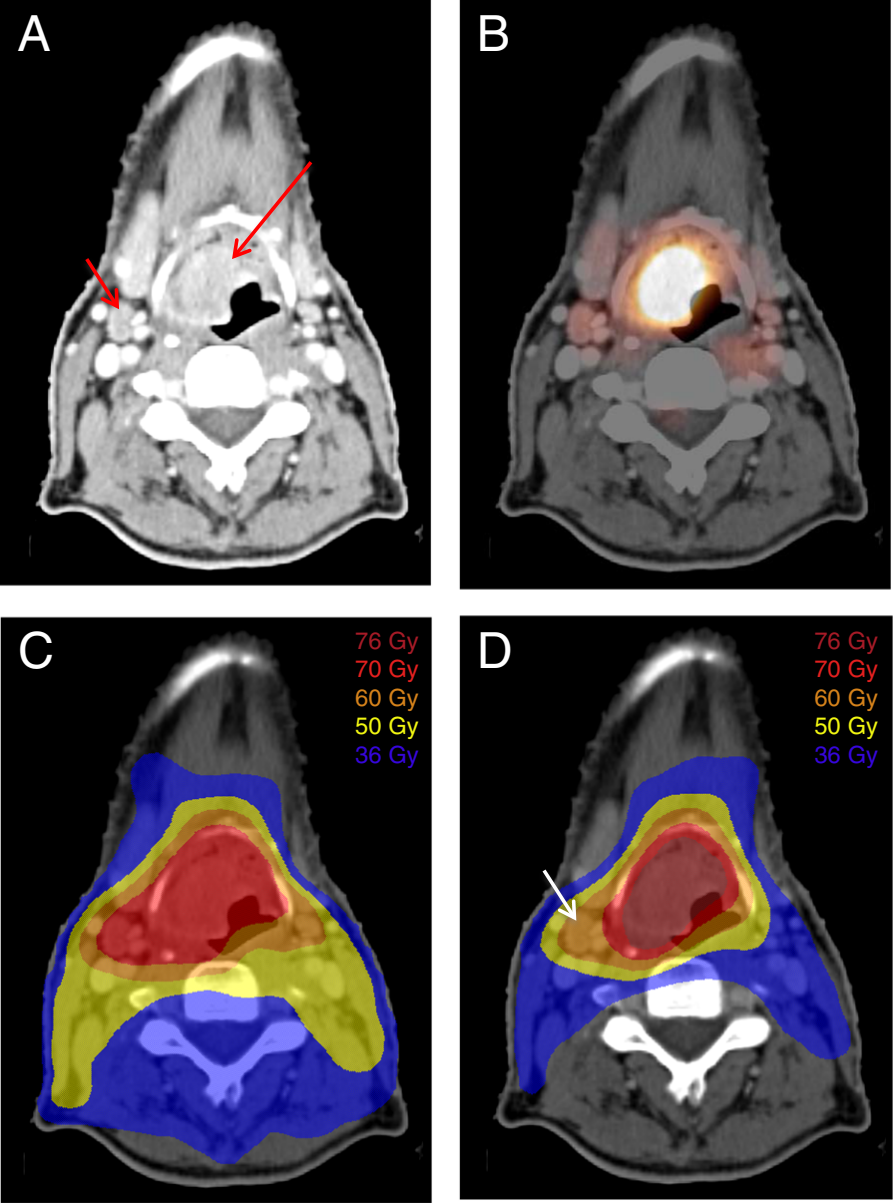
Radiation therapy planning FDG-PET/CT-scan of a patient with an laryngeal squamous cell carcinoma (red arrow) with an intermediate risk lymph node in level 3 right (red arrow) (**a + b**). Comparison of dose planning conform this study protocol for the control-arm (**c**) and intervention-arm (**d**) shows the potential of FDG-PET guided gradient dose prescription with dose reduction to the elective neck in order to better spare organs at risk

To take patient set-up uncertainties into account, a planning target volume (PTV) will be created by extension of the CTV with a 3D margin of 3-5 mm (according to the participating centers protocols). Additionally, for the high-risk tumor volume an additional PTV_BGTV-high-risk_ will be created by extension of the BTV_p_ and GTV_n_ with a 3D margin of 3-5 mm. Differences in target volumes and dose prescription between the treatment arms are illustrated in Fig. 2.

Organs at risk will be delineated according to published international consensus guidelines [21]. Standardized naming of target volumes and organs at risk will facilitate inter-institutional data analysis in this multicenter trial [22].

### Radiation therapy regimen

Patients will be treated with accelerated external beam radiation therapy (EBRT) using volumetric modulated arc therapy (VMAT) or intensity modulated radiation therapy (IMRT) with simultaneous integrated boost (SIB) techniques to deliver multiple dose levels. The total treatment consists of 33 fractions in an overall treatment time of 33 days (5 weeks). During the first 4 weeks of treatment, 6 fractions will be delivered in 5 consecutive days per week. In the last week of treatment, 9 fractions will be delivered (i.e. 4 days bid treatment). The interval between fractions will be at least 6 h. According to the participating centers protocols, offline or online cone beam CT-scans will be made during treatment to verify correct positioning of the patient during irradiation.

Dose prescriptions for the treatment arms are shown in Table 1.

**Table 1:** Dose prescription

Target volume	Dose (fraction dose) (Gy)		^a^EQD2 (Gy)
	Intervention-arm	Control-arm	
PTV_GBTV-high-risk_	66 (2.00)	66 (2.00)	≈ 73
PTV_CTV-high-risk_	62 (1.88)	62 (1.88)	≈ 67
PTV_intermediate-risk_	58 (1.76)	-	≈ 60
PTV_low-risk_	42 (1.27)	48 (1.45)	≈ 35 vs. 45

^a^The equivalent dose in 2 Gy fractions (EQD2) was calculated using the linear-quadratic model using an α/β = 10 Gy for tumor [34]. Differences in treatment time were taken into account by a correction of 0.6 Gy per day to compensate for tumor repopulation [35]

An accelerated fractionation schedule will be used, 33 fractions in 5 weeks (33 days)

### Follow-up

Acute toxicity will be evaluated weekly during treatment and every 2 weeks thereafter, until acute toxicity is completely healed. Acute toxicity will be scored using the common toxicity criteria v2.0 [23]. Standard oncologic follow-up visits are scheduled every 2 months during the 1st year and every 3 months during the 2nd year, after which study participation ends. However, oncologic follow-up will continue every 4 months during the 3rd year and twice annually until at least 5 years of follow-up. During oncologic follow-up visits, late toxicity, recurrence and survival will be evaluated. Late toxicity will be scored using the RTOG/EORTC late radiation morbidity scoring criteria [24].

Periodical study visits will be scheduled once before treatment and at 3, 6, 12 and 24 months after radiation therapy. Subjects will undergo assessment of the swallowing function, thyroid- and salivary glands function and quality of life questionnaires will be completed.

A schedule of study procedures is shown in Table 2.

**Table 2:** Schedule of study procedures

Procedure	Before treatment	During treatment	Months after treatment				
			0	3	6	12	24
Planning FDG-PET/CT-scan	x						
Acute toxicity (CTC v2.0)		x	x	x			
Late toxicity (RTOG-EORTC)				x	x	x	x
Assessment of thyroid function (Blood analysis)	x				x	x	x
Dysphagia related quality of life (PSS-HN, SWAL-QOL)	x			x	x	x	x
Assessment of swallowing function (Water swallowing test)	x			x		x	x
Xerostomia related quality of life (GRIX)	x			x	x	x	x
Assessment salivary gland function (sialometry, sialochemistry)	x			x		x	x
General quality of life (EORTC QLQ-C30, EORTC H&N35)	x			x	x	x	x
Assessment of recurrence			each follow-up visit				

### Assessment of recurrence

If recurrence is suspected during follow-up, additional imaging by MRI, CT, PET or US with FNAC will be performed whatever is judged necessary by the attending physician. Examination under general anesthesia is performed if deemed necessary. All recurrences must be confirmed by cytology or histology.

Central evaluation will be done for all regional recurrences in order to determine if the recurrence occurred in an electively irradiated lymph node. The exact site of recurrence will be reconstructed by performing co-registration of the planning CT-scan with diagnostic imaging of the recurrence.

All recurrences in electively irradiated lymph nodes will be reported as a serious adverse event since this is the safety endpoint of this trial.

### Functional assessments

- *Salivary gland function* will be evaluated in a part of the participating centers only. Stimulated parotid and submandibular salivary flow rates (sialometry) will be measured using techniques described previously [25, 26]. Samples of saliva collected with sialometry will be analyzed for its composition (sialochemistry).

- *Swallowing function* will be evaluated using the water swallowing test [27]. Additional functional performance will be evaluated by the Performance Status Scale for Head & Neck Cancer Patients (PSSH-HN) [16].
- *Thyroid gland function* will be evaluated using standard blood analysis measuring the thyroid stimulating hormone and free thyroxin.

### Assessment of quality of life

For evaluation of general quality of life, the EORTC QLQ-C30 and EORTC QLQ-H&N35 questionnaires will be used [28, 29]. Xerostomia related quality of life will be evaluated using the Groningen Radiation Therapy Induced Xerostomia questionnaire (GRIX) [30] and dysphagia related quality of life will be evaluated using the Swallowing Quality of Life Questionnaire (SWAL-QOL) [31].

### Statistical analysis

All analyses concerning treatment effects will be done according to the intention to treat principle. The student’s t-test will be used to compare ‘normalcy of diet’ scores at 1 year after treatment, the primary endpoint of this trial. A Kaplan-Meier estimate will be calculated for the actuarial rate of recurrence in electively irradiated lymph nodes at 2 years after treatment. Actuarial rates on recurrence and survival will be determined by the date of histopathological diagnosis. Differences between the treatment arms will be assessed using the log-rank test.

For each quality of life questionnaire, data will be included in the analysis if a patient filled in the questionnaire at least at start and at one time-point during the study. Differences in quality of life over time between the intervention- and control arm will be analyzed by using a linear mixed model for repeated measurements. Difference in quality of life scores ≥10 points will be considered clinically relevant [32].

### Safety assurance

After every 5 recurrences in electively irradiated lymph nodes, an interim analysis will be performed following the ‘group sequential approach’ comparing the recurrence rate in the two treatment arms [33]. The *p*-value for the log-rank test statistic will be compared to a nominal α of 0.042 at each interim analysis (i.e. critical value of 1.728) to ensure an overall one-sided α of 0.10 [33]. For this calculation, it is assumed that accrual of 300 patients takes 4 years and that the vast majority of recurrences will be detected within 24 months.

A safety committee will be installed to undertake interim review of the trial safety. The safety committee will consist of an independent statistician and two experienced radiation oncologists in the field of head and neck cancer and will recommend on (dis)continuation of the trial.

### Quality assurance

In order to ensure quality and uniformity between centers, delineation, segmentation and treatment planning guidelines are described in detail in the protocol. The study protocol was discussed with the participating centers until consensus was reached.

Prior to opening inclusion, all participating centers will perform a dummy run in order to assess compliance with the protocols. Also during inclusion, quality assurance by central review will occur prospectively for the first 3 patients included at each participating center, and will occur retrospectively for all included patients thereafter.

## Discussion

### Current status

A total of 6 head and neck centers (or affiliated) will participate and include: the Radboudumc Nijmegen, University Medical Center Utrecht, VU University Medical Center Amsterdam, MAASTRO clinic Maastricht, Radiotherapiegroep Arnhem and Radiotherapeutisch Instituut Friesland.

The first patient was included in august 2016 and accrual is expected to continue for 4 years.

## Abbreviations

BTV: Biological target volume
CT: Computed tomography
CTC: Common toxicity criteria
CTV: Clinical target volume
EANM: European association of nuclear medicine
EARL: EANM research Ltd.
EBRT: External beam radiation therapy
EGFR: Epidermal growth factor receptor
EORTC: European organization for research and treatment of cancer
EQD2: Equivalent dose in 2 Gy fractions
FDG: Fluorodeoxyglucose (18F)
FNAC: fine needle aspiration cytology
GRIX: Groningen radiation therapy induced xerostomia questionnaire
GTV: Gross tumor volume
IMRT: Intensity-modulated radiation therapy
MRI: Magnetic resonance imaging
PET: Positron emission tomography
PSS-HN: Performance status scale for head and neck cancer patients
PTV: Planning target volume
RTOG: Radiation therapy oncology group
SIB: Simultaneous-integrated boost
SUR: Standardized uptake ratio
SUV: Standardized uptake value
SWAL-QOL: Wwallowing quality of life questionnaire
US: Ultrasound
VMAT: Volumetric arc therapy
